# P82 Uncommon culprit: *Campylobacter fetus* infective endocarditis

**DOI:** 10.1093/jacamr/dlaf230.089

**Published:** 2025-12-04

**Authors:** S McLoughlin, A Doherty, C Hickey, T K Keoh, P Stapleton, J Walsh

**Affiliations:** Cork University Hospital, Ireland; Cork University Hospital, Ireland; Cork University Hospital, Ireland; Campylobacter Reference Service in Public Health Laboratory Dublin, Ireland; Cork University Hospital, Ireland; Cork University Hospital, Ireland

## Abstract

**Background:**

*Campylobacter* is a genus of small, spiral Gram-negative rods, commonly associated with foodborne illnesses. Invasive *Campylobacter* infection is a rare clinical entity, associated with substantial risk of mortality. This complication is poorly described because of its rarity, accounting for <1% of *Campylobacter* spp. infections. There is limited published data regarding antimicrobial susceptibility patterns of Campylobacter species and limited interpretive criteria are available. We present a case of *Campylobacter fetus* infective endocarditis and mediastinal collection in a patient with a surgical history of Bentall procedure (combined placement of artificial aortic valve with an ascending aortic prosthetic graft).

**Case presentation:**

: A 72 year old male, who underwent a Bentall procedure 2 years prior, requiring two subsequent sternotomies the following year, presented with fever and an enlarging mass sternal wound, at the site of a previous haematoma. A CT scan of his thorax demonstrated a large mediastinal collection, presumed abscess, extending through the anterior chest wall. Three sets of blood cultures taken on admission were positive for bacterial growth at 39 h; curved Gram-negative bacilli were seen on Gram staining. Subculture confirmed *C. fetus* 2 days later. Vegetations were not evident on transthoracic echocardiogram; the patient declined a transoesophageal echocardiogram. MRI brain showed multiple small infarcts, consistent with embolic events. Based on the Modified Duke criteria, this patient was diagnosed with prosthetic valve infective endocarditis. Radiological findings indicated involvement of the aortic prosthesis and associated mediastinitis. He was initially treated with meropenem, with addition of gentamicin 5mg/kg for the first 11 days. After discussion with colleagues at the *Campylobacter* Reference Service in Public Health Laboratory Dublin, 500 mg oral azithromycin daily, was added. Due to the number of previous redo sternotomies and patient preference, surgical source control was not an option. The large, expanding aortic pseudoaneurysm was deemed a life limiting condition. As the patient wished to be discharged home with palliative care, a life-long suppressive antibiotic was indicated. Ampicillin MIC was 0.75mg/L, however there are no EUCAST or CLSI breakpoints for *C. fetus*. *bla*_OXA-61_ mediated β-lactam resistance has been described in other members of the *Campylobacter* genus but WGS analysis of the isolate at the National Reference Laboratory did not identify any antimicrobial resistance genes. After 7 weeks of IV antibiotics the patient was switched to oral amoxicillin, 1g three times daily, for lifelong suppression and was discharged home, where he remained well after eight months.

**Discussion:**

: Treatment guidelines for *C. fetus* infective endocarditis lack detail on antimicrobial treatment, reflecting limited evidence due to rarity. Consistent with the existing literature, which shows favourable *in vitro* activity and successful clinical outcomes, this case demonstrates successful initial treatment with carbapenem-based therapy. The reference laboratory's genotypic analysis facilitated the de-escalation of antimicrobial therapy, with the absence of resistance gene mechanisms confirming the results of phenotypic testing, providing valuable insights in the setting of limited established antimicrobial breakpoints.

